# Prior-knowledge Fitting of Accelerated Five-dimensional Echo Planar J-resolved Spectroscopic Imaging: Effect of Nonlinear Reconstruction on Quantitation

**DOI:** 10.1038/s41598-017-04065-1

**Published:** 2017-07-24

**Authors:** Zohaib Iqbal, Neil E. Wilson, M. Albert Thomas

**Affiliations:** University of California - Los Angeles, Radiological Sciences, Los Angeles, California 90095 USA

## Abstract

^1^H Magnetic Resonance Spectroscopic imaging (SI) is a powerful tool capable of investigating metabolism *in vivo* from mul- tiple regions. However, SI techniques are time consuming, and are therefore difficult to implement clinically. By applying non-uniform sampling (NUS) and compressed sensing (CS) reconstruction, it is possible to accelerate these scans while re- taining key spectral information. One recently developed method that utilizes this type of acceleration is the five-dimensional echo planar J-resolved spectroscopic imaging (5D EP-JRESI) sequence, which is capable of obtaining two-dimensional (2D) spectra from three spatial dimensions. The prior-knowledge fitting (ProFit) algorithm is typically used to quantify 2D spectra *in vivo*, however the effects of NUS and CS reconstruction on the quantitation results are unknown. This study utilized a simulated brain phantom to investigate the errors introduced through the acceleration methods. Errors (normalized root mean square error >15%) were found between metabolite concentrations after twelve-fold acceleration for several low concentra- tion (<2 mM) metabolites. The Cramér Rao lower bound% (CRLB%) values, which are typically used for quality control, were not reflective of the increased quantitation error arising from acceleration. Finally, occipital white (OWM) and gray (OGM) human brain matter were quantified *in vivo* using the 5D EP-JRESI sequence with eight-fold acceleration.

## Introduction

One of the major goals of *in vivo* ^1^H magnetic resonance spectroscopy (MRS) is to quantify the concentration of metabolites in certain tissues and investigate how these concentrations relate to pathology. Single voxel approaches, such as the point-resolved spectroscopy (PRESS)^1^ sequence, provide a means to quantify several metabolites from a single location. PRESS acquires a one dimensional (1D) spectrum from a volume of interest (VOI), which can subsequently be fit using prior-knowledge 1D fitting algorithms^2–7^, to yield absolute and relative concentration values. Due to severe spectral overlap of certain metabolite resonances in 1D acquisition, techniques have been developed to spread these signals over a second spectral dimension^8–12^, One of these two-dimensional (2D) spectroscopy methods, called J-resolved spectroscopy (JPRESS)^9, 13, 14^, acquires a second spectral dimension by introducing a time increment (*t*_1_) into the PRESS sequence. By acquiring spectra from several *t*_1_ values, JPRESS allows for the detection of both chemical shift in the direct spectral dimension (*F*_2_) and J-coupling in the indirect spectral dimension (*F*_1_). Similar to 1D spectral quantitation, this 2D JPRESS spectrum can be fit using 2D fitting algorithms such as prior-knowledge fitting (ProFit)^15, 16^, to yield metabolite concentrations.

Unfortunately, the main drawback of the JPRESS method is the long acquisition time necessary to obtain the second temporal (spectral) dimension. This becomes an even greater issue when combining JPRESS with spectroscopic imaging (SI)^17, 18^, to obtain metabolic information from multiple spatial dimensions. Several methods have been developed to significantly reduce acquisition time for SI, one of which is the echo planar spectroscopic imaging (EPSI) sequence^19, 20^, EPSI utilizes an echo planar bipolar gradient to simultaneously encode one spatial and one spectral dimension per TR. Different types of k-space readout gradients have also been incorporated into SI in order to accelerate data acquisition^21–24^, Another method capable of reducing scan duration is non-uniform sampling (NUS) combined with compressed sensing (CS) reconstruction^25–27^, CS has been applied to the combined JPRESS and SI method, called J-resolved echo planar spectroscopic imaging (EP-JRESI), for both four dimensional (4D - 2 spatial, 2 spectral)^28^ and five dimensional (5D - 3 spatial, 2 spectral)^29^ acquisitions. In particular, the accelerated 5D EP-JRESI method is capable of up to sixteen-fold acceleration (16x), yielding (*k*_*x*_, *k*_*y*_, *k*_*z*_, *t*_2_, *t*_1_) data in a clinically feasible time. However, the effects of the NUS and iterative, nonlinear reconstruction process on prior-knowledge quantitation results remain unknown. This is especially important with regards to the Cramér Rao lower bounds (CRLBs)^30, 31^, since many investigators utilize these CRLB values for the purposes of analyzing metabolite concentrations. Thus, it is important to determine whether CRLB% values are reflective of the true uncertainty in metabolite concentrations when using accelerated methods.

Therefore, the goal of this study was to investigate the effects of NUS and CS reconstruction on ProFit quantitation of accelerated 5D EP-JRESI data. Synthetic 5D EP-JRESI brain phantom measurements were simulated, and several voxels from these data were then fit using ProFit. Metabolite concentration values, as well as CRLBs, were investigated at several acceleration factors: four-fold (4x), eight-fold (8x), twelve-fold (12x), and 16x acceleration. Additionally, *in vitro* phantom measurements were acquired and were retrospectively undersampled and reconstructed in order to assess the performance of ProFit quantitation. Finally, to demonstrate the application of this method, ProFit was used to quantify occipital white matter (OWM) and gray matter (OGM) voxels from *in vivo* healthy brain measurements obtained using 8x NUS.

## Methods

### 5D EP-JRESI acquisition

The 5D EP-JRESI sequence utilizes both EPSI and NUS to obtain 5D acquisitions, (*k*_*x*_, *k*_*y*_, *k*_*z*_, *t*_2_, *t*_1_) = (16, 16, 8, 256, 64), in a clinically feasible scan time^28, 29^, A fully sampled 5D EP-JRESI acquisition with TR = 1.2 s takes approximately 164 minutes, making NUS a necessity for *in vivo* applications. The sequence was developed by incorporating two major modifications to the standard chemical shift imaging (CSI) method^17^: (1) an echo planar bipolar gradient for readout and (2) a time increment (*t*_1_) inserted between the two 180° pulses. A water acquisition was acquired with only one *t*_1_ point, which was used for eddy current corrections^32^. Water suppression was performed using three WET^33^ pulses for metabolite measurement. Since the echo planar readout gradient is used to acquire the (*k*_*x*_, *t*_2_) dimensions simultaneously, the NUS scheme is applied along the remaining, incremented dimensions: (*k*_*y*_, *k*_*z*_, *t*_1_). The 3D volume produced by (*k*_*y*_, *k*_*z*_, *t*_1_) was sampled according to the following density function based on exponential sampling^34^:

1 $${\mathscr{P}}({k}_{y},{k}_{z},{t}_{1})=exp(-\frac{|{k}_{y}|}{2}-\frac{|{k}_{z}|}{2}-{t}_{1})$$

where ${\mathscr{P}}({k}_{y},{k}_{z},{t}_{1})$ is the probability of sampling a point in the (*k*_*y*_, *k*_*z*_, *t*_1_) volume, and *k*_*y*_, *k*_*z*_, and *t*_1_ were determined by the desired spatial and indirect spectral points, (*k*_*y*_, *k*_*z*_, *t*_1_) = (16, 8, 64): *k*_*y*_ = [−8, −7, −6, …, 7], *k*_*z*_ = [−4, −3, −2, …, 3], and *t*_1_ = [0, 1, 2, …, 64]. This type of non-uniform sampling scheme ensures that points containing higher signal, such as central k-space and earlier *t*_1_ points, are sampled adequately. Sampling masks were generated for each NUS factor (4x, 8x, 12x, and 16x) and were implemented as an option on the console.

Prior to reconstruction, binary filtering was applied, and the spectral range outside 1.2–4.3 ppm in the *F*_2_ dimension was set to zero while the 1.2–4.3 ppm region was unchanged, as previously described^29, 35^, This was performed in order to decrease the dynamic range resulting from residual water/fat signals. In order to reconstruct the missing data points^29^, the following optimization problem was solved in MATLAB using the split Bregman algorithm^36^:

2 $$\mathop{{\rm{\min }}\,TV(u)}\limits_{u}\quad {\rm{s}}{\rm{.t}}.{\Vert R {\mathcal F} u-f\Vert }_{2}^{2} < {\sigma }^{2}$$

**Figure 1:**
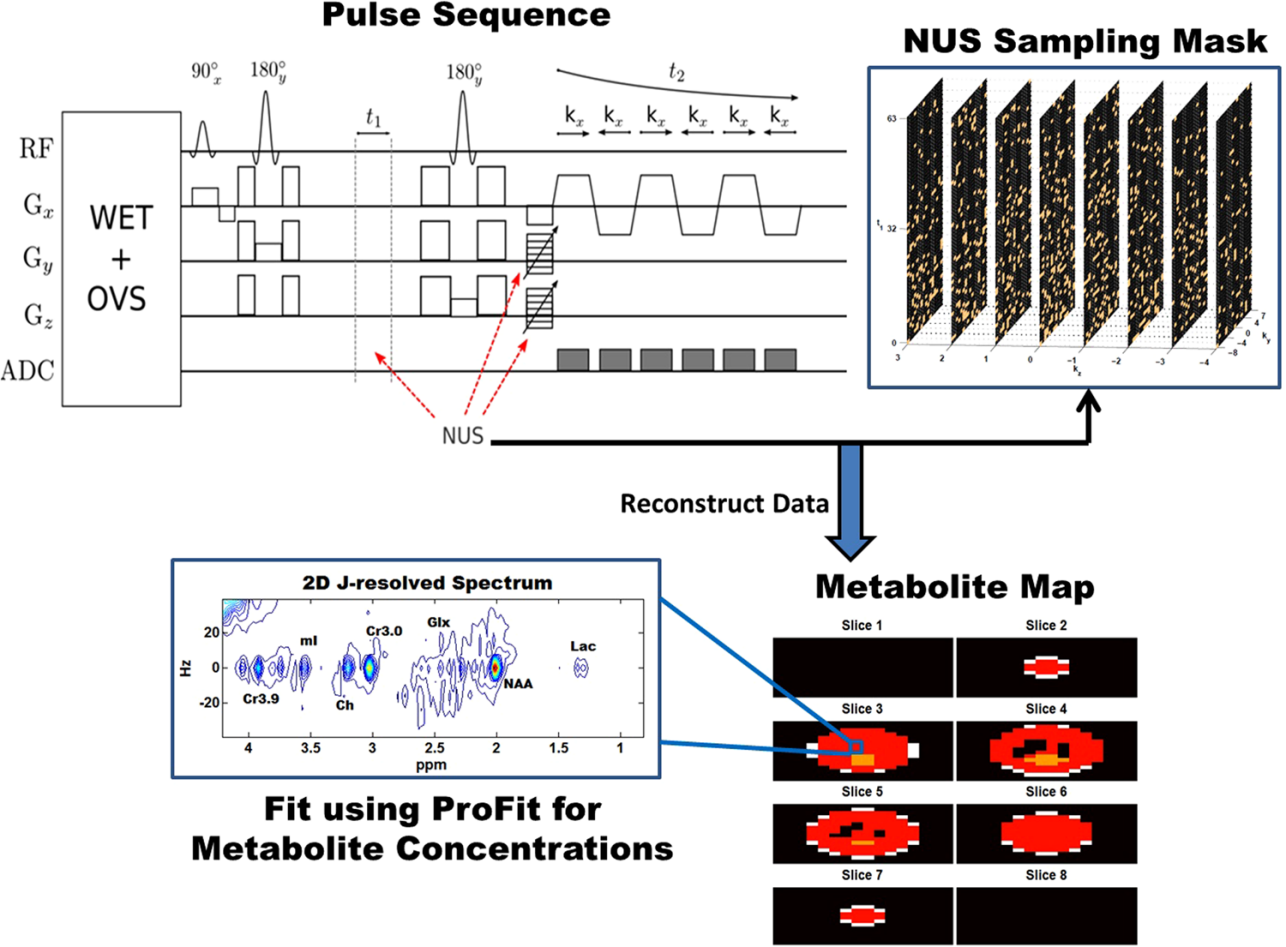
The 5D EP-JRESI acquisition and quantitation methods are shown. First, the data are acquired using the 5D EP-JRESI sequence, which utilizes a 90°–180°–180° pulse sequence for volume localization. The non-uniform sampling scheme is applied to the three incremented dimensions: (*k*_*y*_, *k*_*z*_, *t*_1_), and the copper points are sampled. After acquisition, the data are reconstructed using eq. (2) as described in the text. Following reconstruction, the metabolite maps can be displayed and voxels of interest can be chosen. Finally, these voxels, which contain 2D J-resolved spectra, can be quantified using ProFit to yield metabolite concentrations.

Here, *u* is the reconstructed data, *R* is the sampling mask, ${\mathcal F}$ is the Fourier transform operator applied across the (*k*_*y*_, *k*_*z*_, *t*_1_) dimensions, *f* is the acquired data, and *σ* ^2^ is an estimate of the noise variance. The goal of the optimization was to minimize the total variation (TV) of the reconstructed data, and was implemented on a coil-by-coil basis. Here, TV is applied on the three undersampled dimensions, (*y*, *z*, *t*_1_). The reconstruction took approximately 30–45 minutes per coil on a workstation equipped with a 3.1 GHz Intel Xeon dual processor and 128 GB RAM. Finally, coil combination was performed after applying frequency drift and phase corrections as previously described^29, 37^. A general schematic for the accelerated 5D EP-JRESI acquisition method can be seen in Fig. 1.

### ProFit quantitation

For this study, the new version of ProFit^16^ was used to fit the 2D J-resolved spectra obtained using the 5D EP-JRESI technique. ProFit uses non-linear least squares fitting to fit an actual spectrum using prior-knowledge spectra obtained either experimentally or through simulation. The degrees of freedom for fitting are increased from iteration to iteration, allowing for measurement of line-broadening factors, frequency shifts, concentrations, and other parameters for several metabolites. First, a basis set was created using GAMMA simulation^38^ and previously reported chemical shifts and J-coupling constants^39^ of 22 metabolites: creatine 3.0 (Cr3.0), creatine 3.9 (Cr3.9), n-acetyl aspartate (NAA), phosphorylcholine (PCh), choline (Ch), glycerylphosphoryl choline (GPC), aspartate (Asp), *γ*-aminobutyric acid (GABA), glucose (Glc), glutamine (Gln), glutamate (Glu), glutathione (GSH), lactate (Lac), myo-inositol (mI), n-acetyl aspartyl glutamate (NAAG), phosphoethanolamine (PE), taurine (Tau), threonine (Thr), scyllo-inositol (Scy), alanine (Ala), glycine (Gly), and ascorbic acid (Asc). Small modifications were made to the fitting algorithm: (1) only three of the four outer iterations were used, foregoing spline baseline modeling and (2) the inner iterations were limited to decrease fitting time. The second modification was accomplished by simply changing the maximum number of iterations that the lsqnonlin function in MATLAB used in the optimization options. In addition to concentration values, ProFit also calculates the CRLB values for each metabolite^30, 31^, from the Fisher matrix:

3 $$F=\frac{Re( {\mathcal B} ^{\prime} \ast  {\mathcal B} )}{{\sigma }^{2}}$$

Eq. (3) uses the basis matrix, ${\mathcal B}$, and an estimate of noise variance, *σ* ^2^, to yield the Fisher matrix (F). ${\mathcal B}$ is a vectorization of all the 2D spectra in the basis set, and therefore each row in ${\mathcal B}$ corresponds to the prior-knowledge of each metabolite in the (*F*_2_,*F*_1_) domain. Noise variance is estimated in ProFit from a spectral region far removed from the diagonal peaks. CRLB values for the metabolites are readily obtained from the following:

4 $$CRLB=\sqrt{diag({F}^{-1})}$$

The diagonal elements from *F* ^−1^, denoted as *diag*(), yield CRLB values for each individual metabolite. Relative CRLBs, CRLB%, can be obtained by dividing the CRLB by the corresponding metabolite concentration and multiplying by 100:

5 $$CRLB \% =100(\frac{CRLB}{conc})$$

Typically, 20–30% is the acceptable CRLB% used in a variety of *in vivo* studies.

### Virtual Phantoms

A three dimensional Shepp-Logan brain phantom was simulated to replicate the 5D EP-JRESI experiment with the following scan parameters: *B*_0_ = 2.89 T (123.23 MHz), (*k*_*x*_, *k*_*y*_, *k*_*z*_, *t*_2_, *t*_1_) = (16, 16, 8, 256, 64), direct spectral bandwidth = 1190 Hz, indirect spectral bandwidth = 500 Hz, and TE = 30 ms. The two-dimensional spectra were simulated using GAMMA simulation^38^ with concentration values similar to previously reported *in vivo* values^39^. The virtual phantom, or vphantom, included NAA (10.5 mM), NAAG (1.7 mM), GABA (1.3 mM), Ala (0.27 mM), Asc (0.6 mM), Asp (3.4 mM), Ch (0.25 mM), PCh (0.8 mM), GPC (2 mM), Cr (8 mM), Glc (0.3 mM), Glu (11.3 mM), Gln (5.3 mM), GSH (2.3 mM), mI (6 mM), Lac (1.8 mM), PE (1.6 mM), Scy (0.6 mM), Tau (2.7 mM), and Thr (0.5 mM). The signal intensities of the spectra were modulated based on the spatial structure of the Shepp-Logan phantom.

After simulation, the vphantom was retrospectively undersampled using the NUS schemes for 4x, 8x, 12x, and 16x acceleration. These data were subsequently reconstructed to produce accelerated 5D EP-JRESI data, and seventy-six voxels from each vphantom measurement were quantified using ProFit. Metabolite means, standard deviations, CRLB% values, and normalized root mean square error (nRMSE) values were investigated using these 76 voxels. The nRMSE was calculated using the fully sampled vphantom measurement as the true value:

6 $$nRMSE=\frac{\sqrt{\frac{1}{n}\sum _{i=1}^{n}{({c}_{f,n}-{c}_{us,n})}^{2}}}{{\bar{c}}_{f}}$$

where *c*_*f*,*n*_ is the ProFit result from the fully sampled vphantom measurement, *c*_*us*,*n*_ is the ProFit result from the undersampled vphantom measurement, *n* is the number of voxels (*n* = 76), and ${\bar{c}}_{f}$ is the average ProFit result from the fully sampled vphantom. Considering typical *in vivo* metabolite standard deviations, an error of 15% was chosen as the cutoff for acceptable quality. In order to examine the spatial effects of the reconstruction, metabolite maps were produced using peak integration for NAA (1.9–2.1 ppm) and mI (3.4–3.6 ppm). Eq. (6) was applied voxel by voxel to calculate nRMSE for both the NAA and mI spatial maps.

Additionally, a second vphantom was simulated in order to investigate the effects of NUS and reconstruction on the spatial signal leakage more carefully. The second vphantom was simulated with the same metabolite concentrations described above, however signal was only placed in a 3 × 3 voxel region in the central slice (slice 5). The second vphantom underwent the same undersampling and reconstruction process that was described for the first vphantom. The spatial leakage from the 3 × 3 region was assessed both qualitatively and quantitatively by measuring the NAA and Asp signals across the (*y*,*z*) dimensions.

### *In Vitro* Phantom

Nine gray matter brain phantom measurements were obtained using the fully sampled 5D EP-JRESI sequence on a Siemens 3 T Trio (Siemens Healthcare, Erlangen, Germany) scanner. The phantom contained the following metabolites: NAA(8.9 mM), NAAG(0.51 mM), GABA(0.7 mM), Asp(2.1 mM), Ch(0.9 mM), PCh(0.6 mM), Cr(7 mM), Glc(1 mM), Glu(12.5 mM), Gln(2.5 mM), GSH (2 mM), mI(4.4 mM), Lac(1 mM), PE(1 mM), Tau(1.8 mM), and Thr(0.3 mM). The following scan parameters were used for the 5D EP-JRESI acquisition: field of view (FOV) = 16 × 16 × 12 cm^3^, (*k*_*x*_, *k*_*y*_, *k*_*z*_, *t*_2_, *t*_1_) = (16, 16, 8, 256, 64), direct spectral bandwidth = 1190 Hz, indirect spectral bandwidth = 500 Hz, and TR/TE = 1200/30ms. Retrospective undersampling using the NUS schemes for 4x, 8x, 12x, and 16x acceleration was performed for each phantom and these data were subsequently reconstructed to produce accelerated 5D EP-JRESI data. Eight voxels were chosen from each phantom measurement, and were quantified using ProFit for all of the acceleration factors used. Metabolite means, standard deviations, and CRLB% values were investigated using these 72 voxels.

### *In Vivo*

Ten healthy volunteers (mean age = 25 years old) were scanned using the 8x accelerated 5D EP-JRESI sequence prospectively on a Siemens 3 T Trio (Siemens Healthcare, Erlangen, Germany) scanner. Outer volume suppression (OVS) bands were applied to suppress skull marrow lipids and contaminating signal outside the PRESS localization in addition to global water suppression^33^ for all *in vivo* measurements. The acquisitions were performed using the same parameters listed for the vphantom and *in vitro* measurements. Additionally, the TR used for acquisition was 1200 ms, the field of view (FOV) was set to 24 × 24 × 12 cm^3^, and the PRESS VOI was changed for each volunteer but was typically 9 × 12 × 6 cm^3^. Occipital white matter (OWM) and gray matter (OGM) voxels were identified for each volunteer and were fit using ProFit. In all cases, the medial occipital gray matter region was first identified, and then the voxel left of the OGM was assumed to be the OWM. Metabolite means and standard deviations were used to compare the metabolite concentrations for the two brain regions.

### Statistical Analysis

For the virtual phantoms, all errors were calculated using standard nRMSE calculations as described in Eq. (6). However, the differences between the retrospective *in vitro* phantom measurements were assessed using means and standard deviations of the ProFit results. Furthermore, a Student’s t-test was used to determine if any significant (p < 0.05) differences were found between the fully sampled and accelerated phantom data for each metabolite. Similarly, multiple Student’s t-tests were also used to compare the OGM and OWM voxels from the *in vivo* measurements. To be more stringent when analyzing the *in vivo* results, a Bonferroni correction^40^ was applied, and significance was therefore determined to be p < 0.004.

## Results

### Virtual Phantoms

**Figure 2:**
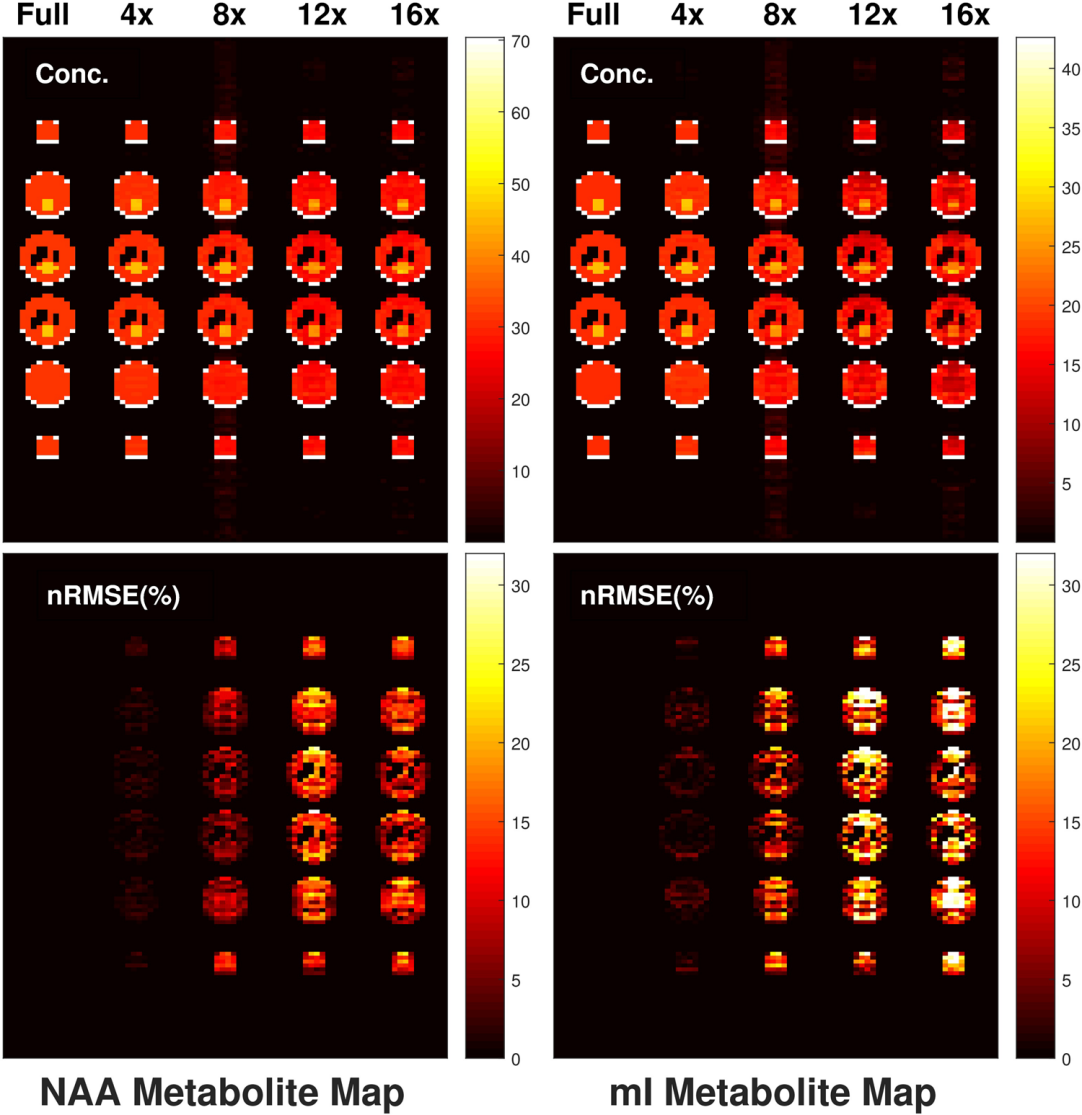
Metabolite maps are displayed for NAA (left) and mI (right). Concentration maps (top) as well as nRMSE maps (bottom) demonstrate the overall depreciation of accuracy as fewer points are sampled before reconstruction. Higher error is prevalent in the central part of the phantom when compared to the left and right edges. At 8x, NAA had a spatial nRMSE% value of 7%, whereas mI had a spatial nRMSE% value of 11%.

Metabolite maps of NAA and mI can be seen in Fig. 2 for the fully sampled simulation, and also for the 4x, 8x, 12x, and 16x accelerated vphantoms. The average nRMSE values for NAA were 1.34% for 4x, 6.47% for 8x, 13.4% for 12x, and 11.6% for 16x acceleration. Similarly, the average nRMSE values for mI were 2.07% for 4x, 10.6% for 8x, 18.7% for 12x and 20.6% for 16x acceleration. The reconstructed signal error increased throughout the entire vphantom as the acceleration factor increased, as demonstrated by the higher nRMSE values.

**Figure 3:**
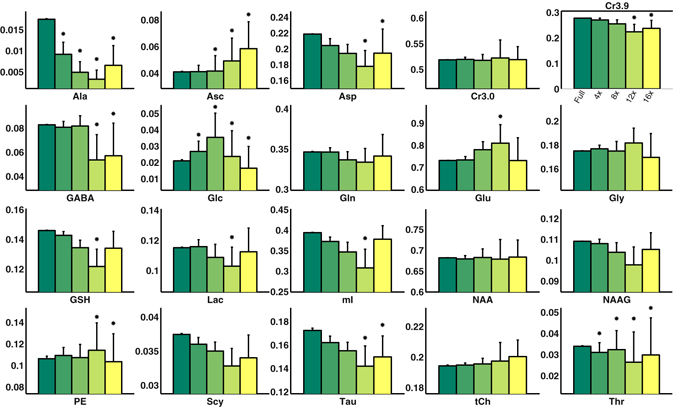
Bar graphs comparing the fully sampled ProFit quantitation results with the 4x, 8x, 12x, and 16x accelerated quantitation results are shown for the vphantom. An asterisk (*) is indicative of normalized root mean square error that exceeded 15% when compared to the fully sampled phantom for the 76 voxels. Most metabolites did not exceed the nRMSE threshold until 12x acceleration, with the exception of Ala, Asc, Glc, and Thr. Higher concentrated metabolites, such as NAA, Cr3.0, and tCh, did not show nRMSE% values higher than 15% even at 16x acceleration.

**Figure 4:**
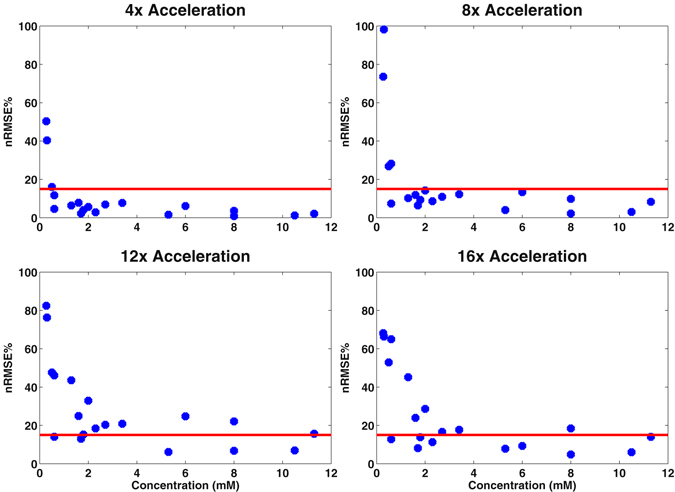
Concentration values for the simulated metabolites are plotted against nRMSE% values in blue. The red line is indicative of nRMSE% = 15%, which is the cutoff for acceptable quality used in this study. From the plots, it is apparent that nRMSE% increases as the metabolite concentration decreases. Furthermore, nRMSE% increases as the acceleration factor increases in general.

ProFit quantitation of 76 voxels from the fully sampled and accelerated 5D EP-JRESI vphantom acquisitions yielded several important findings. Figure 3 shows a bar graph with the concentration values for the metabolites fit using ProFit. The standard deviations for these concentration values increased as the acceleration factor increased. Most of the metabolites did not exceed the nRMSE threshold of 15% until 12x acceleration. However, metabolites that were simulated with lower concentrations such as Ala, Asc, Glc, and Thr exhibited nRMSE values greater than 15% at 4x and 8x acceleration. Figure 4 shows the nRMSE% values as a function of simulated concentration values (mM) to better highlight this result. The mean CRLB% values did not exceed 20% for most metabolites even at 16x acceleration, as seen in Table 1. However, the standard deviation of the CRLB% values for the metabolites steadily increased as the acceleration factor increased. At 4x acceleration, the standard deviation for the CRLB% values for most metabolites was 20% of the mean CRLB% value, whereas for 8x it was 40%, for 12x it was 80%, and for 16x acceleration it was also 80%.

**Table 1:** The mean ± standard deviation CRLB values and normalized root mean square errors comparing the fully sampled and accelerated 5D EP-JRESI vphantom measurements are tabulated as percents.

Metabolite	Full		4x		8x		12x		16x	
	CRLB (%)	nRMSE (%)	CRLB (%)	nRMSE (%)	CRLB (%)	nRMSE (%)	CRLB (%)	nRMSE (%)	CRLB (%)	nRMSE (%)
Ala	2.17 ± 0.02	—	6.11 ± 2.63	50.31	13.7 ± 9.21	73.54	39.2 ± 26.3	82.39	17.2 ± 14.8	68.12
Asc	2.15 ± 0.02	—	2.84 ± 0.57	11.73	3.65 ± 3.44	28.19	8.26 ± 7.98	46.05	4.03 ± 2.74	64.97
Asp	0.49 ± 0.00	—	0.69 ± 0.14	7.73	0.80 ± 0.32	12.27	2.62 ± 1.83	20.83	1.46 ± 1.00	17.72
Cr3.0	0.04 ± 0.00	—	0.05 ± 0.01	0.83	0.06 ± 0.02	2.19	0.17 ± 0.12	6.76	0.10 ± 0.05	4.83
Cr3.9	0.13 ± 0.00	—	0.18 ± 0.04	3.60	0.21 ± 0.08	9.83	0.62 ± 0.47	22.08	0.34 ± 0.20	18.41
GABA	0.73 ± 0.00	—	0.99 ± 0.21	6.37	1.09 ± 0.44	10.24	7.38 ± 8.34	43.55	4.05 ± 4.29	45.13
Glc	7.25 ± 0.15	—	7.99 ± 2.83	40.36	7.90 ± 6.44	98.22	28.5 ± 23.5	76.27	35.3 ± 37.8	66.35
Gln	0.24 ± 0.00	—	0.32 ± 0.06	1.59	0.36 ± 0.14	3.99	1.07 ± 0.77	6.14	0.61 ± 0.33	7.81
Glu	0.08 ± 0.00	—	0.11 ± 0.02	2.09	0.12 ± 0.05	8.29	0.36 ± 0.26	15.64	0.22 ± 0.13	14.01
Gly	0.25 ± 0.00	—	0.33 ± 0.07	1.99	0.37 ± 0.15	4.59	1.02 ± 0.72	8.04	0.68 ± 0.43	11.77
GPC	0.29 ± 0.00	—	0.38 ± 0.08	5.63	0.40 ± 0.15	14.21	0.98 ± 0.74	32.85	0.61 ± 0.35	28.60
GSH	0.23 ± 0.00	—	0.31 ± 0.06	2.84	0.36 ± 0.14	8.61	1.21 ± 0.89	18.42	0.63 ± 0.35	11.27
Lac	0.91 ± 0.00	—	1.18 ± 0.25	4.05	1.37 ± 0.54	9.34	4.20 ± 2.82	15.22	2.34 ± 1.43	13.83
mI	0.15 ± 0.00	—	0.21 ± 0.04	6.11	0.24 ± 0.10	13.37	0.77 ± 0.56	24.70	0.44 ± 0.28	9.27
NAA	0.03 ± 0.00	—	0.04 ± 0.01	1.18	0.04 ± 0.02	3.01	0.13 ± 0.09	6.91	0.08 ± 0.04	5.99
NAAG	0.19 ± 0.00	—	0.25 ± 0.05	2.19	0.29 ± 0.12	6.41	0.93 ± 0.66	13.00	0.51 ± 0.29	8.12
PE	1.23 ± 0.03	—	1.58 ± 0.34	7.82	1.76 ± 0.70	11.88	4.85 ± 3.50	24.90	3.34 ± 2.19	23.93
Scy	0.43 ± 0.00	—	0.59 ± 0.12	4.61	0.67 ± 0.26	7.39	2.13 ± 1.62	14.04	1.19 ± 0.68	12.79
Tau	0.58 ± 0.01	—	0.81 ± 0.17	6.88	0.93 ± 0.35	10.91	3.13 ± 2.30	20.32	1.75 ± 1.10	16.59
tCh	—	—	—	0.79	—	1.96	—	6.43	—	6.32
Thr	3.37 ± 0.04	—	4.99 ± 1.43	16.05	5.77 ± 3.82	26.83	17.69 ± 14.71	47.59	13.7 ± 15.4	52.88

Many CRLB% values remain well below the 20% threshold typically used for quality control.

**Figure 5:**
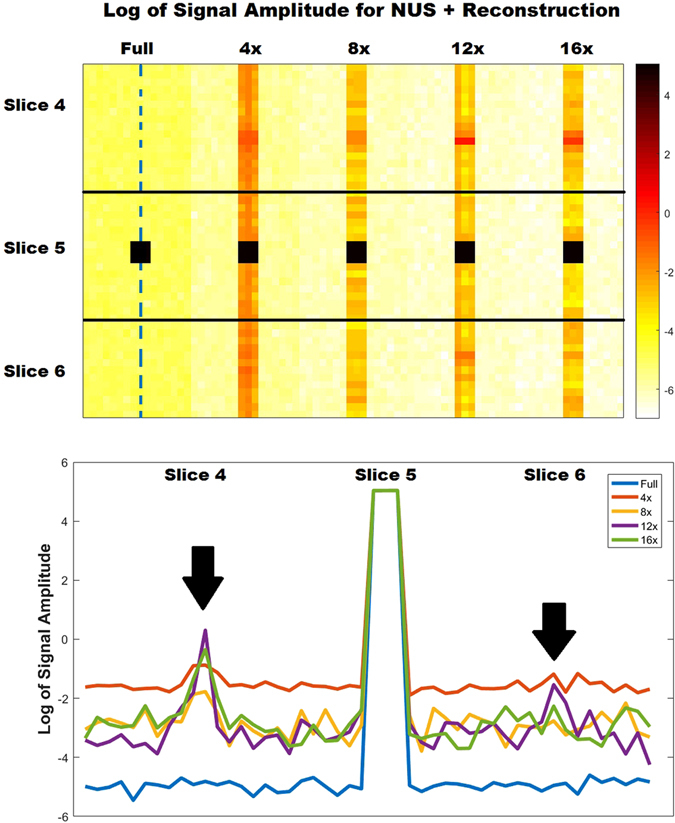
The virtual phantom results simulated with signal only in a 3 × 3 voxel region in the central slice (slice 5) are displayed. This vphantom demonstrates the signal leakage as a result of each non-uniform sampling scheme and reconstruction. The log of the NAA signal amplitudes are shown for the spatial display (top). Additionally, the profile shown by the blue dashed line is displayed for each NUS and reconstruction simulation (bottom). From the signal profiles, it is clear that there are signal leakages in the neighboring slices when utilizing 12x and 16x acceleration (black arrows). Surprisingly, 4x results have a uniform leakage of signal throughout the phase-encoded and slice direction due to less denoising. Signal leakage was generally lower than the true signal by 4 orders of magnitude for all of the acceleration factors.

Figure 5 shows the results of the second vphantom study. Three slices (slice 4 - slice 6) are shown for the fully sampled simulation, as well as the simulated results using NUS and non-linear reconstruction for 4x, 8x, 12x, and 16x acceleration. While there is signal leakage present for all of the accelerations, the maximum of this signal leakage is less than 1% of the original signal. While only the NAA results are shown, the signal leakage is still under 1% for much lower concentrated resonances as well, including Asp and mI. The leakage is exclusive to the undersampled spatial dimensions, (*y*,*z*), and is more apparent in the *z* direction, which is exemplified in Fig. 5.

### *In Vitro* Phantom

**Figure 6:**
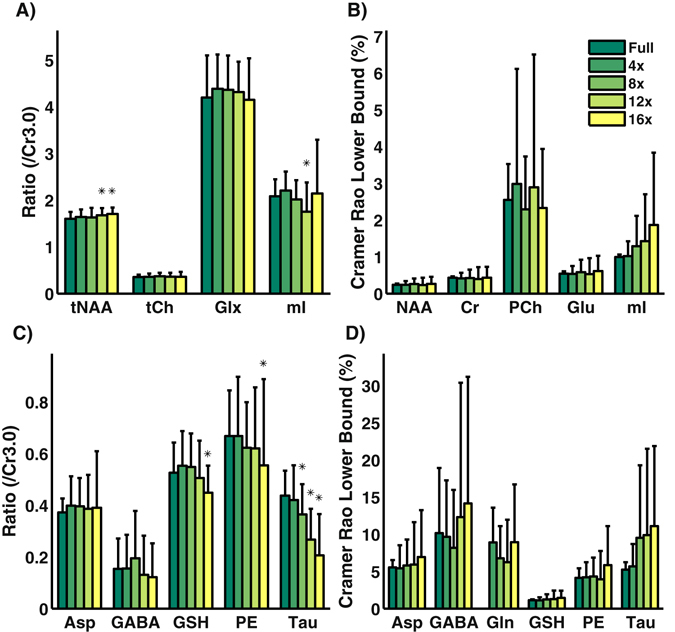
Seventy-two voxels (n = 72) acquired over nine phantom measurements were quantified using ProFit. Phantom measurements were retrospectively undersampled to achieve four acceleration factors (4x, 8x, 12x, and 16x) and were reconstructed before quantitation. Metabolite ratios with respect to Cr3.0 (**A** and **C**) and Cramér Rao Lower Bound values (**B** and **D**) are shown. For metabolite ratios, an asterisk denotes a significant difference (p < 0.05) between the accelerated and the fully sampled results using a student’s t-test. With the exception of Tau, most of the significant differences appear after 8x acceleration.

Concentrations and CRLB% values for several metabolites calculated from the 5D EP-JRESI data acquired in the phantom can be seen in Fig. 6. For quantitation purposes, certain metabolites were combined: tNAA (NAA + NAAG), tCh (Ch + PCh + Gpc), and Glx (Glu + Gln), similar to what has been widely practiced in 1D MRS. This combination was performed because ProFit would over-estimate or under-estimate some of these metabolite components, resulting in values with very high standard deviations. This over/under estimation was present in both fully sampled and accelerated measurements. Due to the binary filtering before reconstruction, Lac was not reliably quantified. The average CRLB% values remained relatively low, and only exceeded the cutoff of 30% for GABA and Tau. Using 8x acceleration yielded no significant differences from the fully sampled results, except for Tau. However, several significant differences did exist for 12x and 16x NUS.

### *In Vivo*

**Figure 7:**
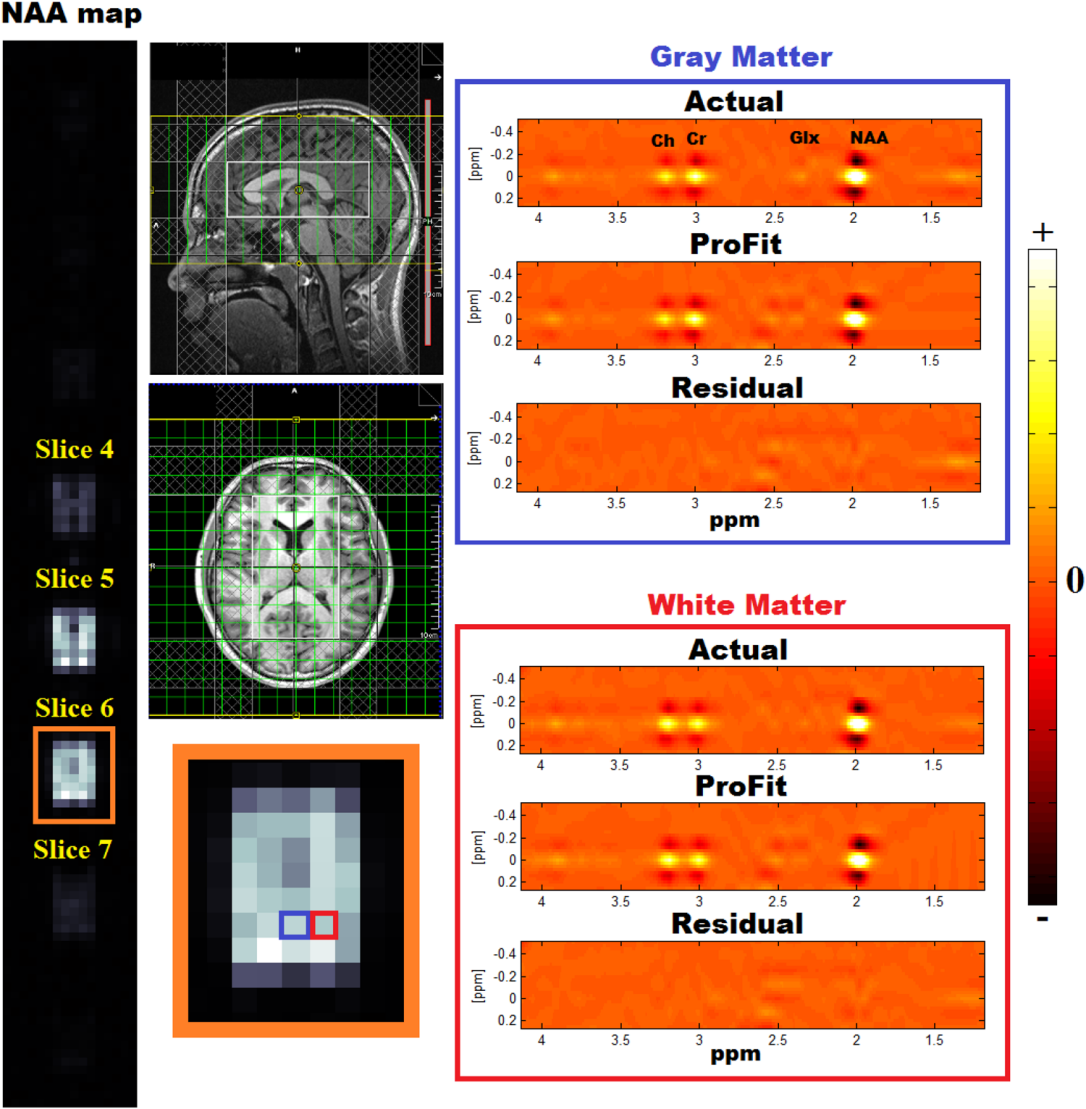
The NAA metabolite map from a healthy adult (age = 20 years old) is shown (left) alongside sagittal and axial *T*_1_-weighted MRI (middle). Two voxels from slice 6 were identified as occipital gray matter (blue) and white matter (red). These two voxels were fit using ProFit (right), and the real part of the spectrum is displayed. Due to the mixed phase of the peaks, negative side-lobes are apparent for the singlets NAA, Cr, and Ch. Fit residuals for both voxels are very low, demonstrating the reliable fitting of ProFit.

**Figure 8:**
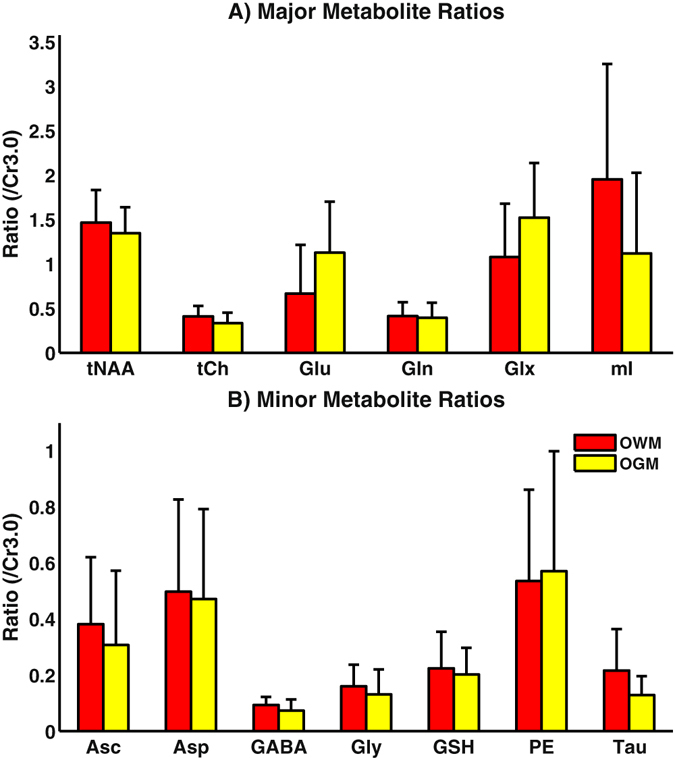
Occipital white matter (red) and gray matter (yellow) voxels were identified and quantified using ProFit for ten healthy adults (mean age = 25 years old). Metabolite ratios with respect to Cr3.0 are displayed for higher concentration (**A**) and lower concentration (**B**) metabolites. Although no significant (p < 0.004) differences were seen between the two spatial regions, expected trends for tNAA, Glx, and mI are observed.

An example *in vivo* 5D EP-JRESI data set can be seen in Fig. 7. A metabolite map of NAA, which was produced by displaying the signal intensity calculated using peak integration over three spatial dimensions, is shown. Signal is only present in 3–4 slices due to the choice of the VOI and FOV. The fit results using ProFit show low residuals for both the OGM and OWM. The mean values and standard deviations over all ten healthy volunteers can be seen in Fig. 8.

## Discussion

From the vphantom, *in vitro*, and *in vivo* results presented, it is clear that the ProFit algorithm is capable of fitting 2D J-resolved spectra acquired using the accelerated 5D EP-JRESI method. The vphantom results demonstrated nRMSE values of less than 15% for most metabolites when using 4x and 8x acceleration. NAA, Cr3.0, and tCh, which were both simulated with higher concentrations, had nRMSE values less than 7% when using 16x acceleration. The signal leakage for all metabolites was less than 1% of the original signal, as determined by the results from the second vphantom. Additionally, the retrospective *in vitro* phantom measurements demonstrated significant differences (p < 0.05) for several metabolites at 12x and 16x acceleration. For the *in vivo* measurements, voxels were chosen containing a majority of either white or gray matter consistent with a reference *T*_1_-weighted MRI. Due to the spatial resolution of 1.5 × 1.5 × 1.5 cm^3^, quantified OWM and OGM voxels were not purely white or gray matter, but were instead a mixture of the two. This could be the major reason why no significant differences were found between the two spatial regions. Nonetheless, ProFit results from the 8x accelerated 5D EP-JRESI in healthy volunteers demonstrated metabolic trends for white and gray matter that were consistent with previous literature values^41^: elevated tNAA, tCh, and mI in the OWM, and elevated Glx in the OGM. These findings show the potential of ProFit quantitation in combination with the accelerated 5D EP-JRESI acquisition to differentiate tissues *in vivo*.

Spatially, higher nRMSE values were present as the acceleration factor increased. For the 8x accelerated metabolite maps displayed in Fig. 2, mI was simulated at 6 mM and had a spatial nRMSE of 10.6%, whereas NAA was simulated at 10.5 mM and had a spatial nRMSE of only 6.5%. This implies that lower concentration metabolites will have even larger spatial profile errors at 8x acceleration. The 12x and 16x reconstructions suffered from large errors in the central regions of the phantom. From Fig. 5, it is clear that there is minimal signal leakage present within the same slice, however this leakage is increased in neighboring slices. This is primarily due to the fact that *y* = 16, while *z* = 8 for the acquisition. Since *y* has more points, signal leakage or aliasing along this dimension is suppressed, while signal aliasing is more problematic in the *z* dimensions because of the limited number of points. The *in vivo* NAA metabolite map shown in Fig. 7 does not show noticeable aliasing artifacts, however. Therefore, for accurate spatial reconstruction, 8x acceleration appears to be the limit for this simulated brain phantom.

Both the vphantom and *in vitro* phantom measurements demonstrated that higher acceleration factors (12x and 16x) increased concentration error results as quantified by ProFit for several metabolites: Ala, Asc, Asp, Cr3.9, GABA, Glc, Glu, GSH, Lac, mI, PE, Tau, and Thr. Furthermore, lower concentration metabolites yielded higher errors even when using 4x and 8x acceleration. Since these signals were present very close to the spectral baseline, the reconstruction method was not capable of faithfully reconstructing these metabolites. In general, metabolites that had concentrations greater than 0.5 mM were accurate (nRMSE < 15%) until 12x acceleration.

Surprisingly, CRLB% values did not change drastically for most metabolites, even at 16x acceleration. From Eqs (3) and (4), it can be shown that the relationship between CRLB and noise standard deviation is: CRLB ∝ *σ*. Since the CRLB% did not change drastically, it is implied that noise was accurately reconstructed within the 2D spectra. From Table 1, it is clear that CRLB% is not a good indicator of reconstruction error, since several metabolites with nRMSE values greater than 15% would pass the CRLB% < 20% criterion. The CRLB% metric is more indicative of the orthogonality between basis sets for the metabolites, and using this as a quality control metric for ProFit quantitation of 2D J-resolved spectra from the accelerated 5D EP-JRESI acquisition may not be appropriate. In fact, using the CRLB% value as a quality filtering metric introduces bias into the quantitative results^42, 43^.

Aside from the NUS and iterative, nonlinear reconstruction process, other scan parameters and processing methods are also important for *in vivo* ProFit quantitation of the 5D EP-JRESI data. In order to shorten the acquisition duration, a TR of 1200ms was chosen as one of the scan parameters. This choice led to *T*_1_ weighting of the spectra, which is a common problem in MRSI with one spectral dimension as well, and affected *in vivo* quantitation of metabolites^44^. Another scan parameter, *t*_2_, may have also played a role in quantitation results. ProFit may have been able to distinguish metabolites with greater ease if spectral resolution was improved. However, acquiring more *t*_2_ points leads to larger data sizes, greatly increasing the amount of memory necessary for reconstruction. With higher computational capabilities, however, *t*_2_ = 512 or 1024 may be more feasible. In addition to WET pulses and OVS bands, which were used to suppress water and lipid signals, binary filtering was also applied to the direct spectral domain before reconstruction. This made it difficult to quantify metabolites close to or outside the 1.2–4.3 ppm range, such as Lac. With improvements to the sequence design, including better water^45^ and fat^46^ suppression pulses, or perhaps including metabolite cycling methods^47–49^, other metabolites may be quantified using ProFit *in vivo*.

Finally, taking into consideration the current scan parameters and processing methods, the vphantom, *in vitro* and *in vivo* results suggest that 8x acceleration is the upper limit for decreasing scan time while retaining accurate concentration values from ProFit. Metabolite ratios quantified *in vivo* as less than 0.06 with respect to Cr3.0 may have significant error even if the reported CRLB% is less than 20%. Application of ProFit quantitation to different accelerated 2D spectral SI methods, such as the accelerated echo planar correlated spectroscopic imaging (EP-COSI) method^50–52^, still needs to be evaluated. Utilization of higher acceleration factors may be achieved for different applications, including muscle and breast SI, since the primary resonances of interest (fat) have higher concentrations *in vivo*. Future studies will focus on implementing the 8x accelerated 5D EP-JRESI sequence in different pathologies and quantifying the results using ProFit.

## Conclusion

This study demonstrates that the ProFit algorithm can be used to quantify 2D J-resolved spectra obtained from accelerated 5D EP-JRESI acquisitions. With the current implementation of the sequence, 8x acceleration is the upper limit for NUS while retaining accurate (nRMSE < 15%) ProFit quantitation results for many metabolites. Furthermore, quantitation of occipital white and gray matter regions in the brain using ProFit demonstrated regional trends consistent with previous *in vivo* results.
